# Combination of Intratumoral Invariant Natural Killer T Cells and Interferon-Gamma Is Associated with Prognosis of Hepatocellular Carcinoma after Curative Resection

**DOI:** 10.1371/journal.pone.0070345

**Published:** 2013-08-05

**Authors:** Yong-Sheng Xiao, Qiang Gao, Xiang-Nan Xu, Yi-Wei Li, Min-Jie Ju, Ming-Yan Cai, Chen-Xin Dai, Jie Hu, Shuang-Jian Qiu, Jian Zhou, Jia Fan

**Affiliations:** 1 Liver Cancer Institute, Zhongshan Hospital, Fudan University, Shanghai, P. R. China; 2 Key Laboratory for Carcinogenesis and Cancer Invasion, Ministry of Education, Shanghai, P. R. China; Johnson & Johnson Medical, China

## Abstract

**Purpose:**

To investigate the prognostic value of intratumoral invariant natural killer T (iNKT) cells and interferon-gamma (IFN-γ) in hepatocellular carcinoma (HCC) after curative resection.

**Experimental Design:**

Expression of TRAV10, encoding the Vα24 domain of iNKT cells, and IFN-γ mRNA were assessed by quantitative real-time polymerase chain reaction in tumor from 224 HCC patients undergoing curative resection. The prognostic value of these two and other clinicopathologic factors was evaluated.

**Results:**

Either intratumoral iNKT cells and IFN-γ alone or their combination was an independent prognostic factor for OS (*P* = 0.001) and RFS (*P* = 0.001) by multivariate Cox proportional hazards analysis. Patients with concurrent low levels of iNKT cells and IFN-γ had a hazard ratio (HR) of 2.784 for OS and 2.673 for RFS. The areas under the curve of iNKT cells, IFN-γand their combination were 0.618 vs 0.608 vs 0.654 for death and 0.591 vs 0.604 vs 0.633 for recurrence respectively by receiver operating characteristic curve analysis. The prognosis was the worst for HCC patients with concurrent low levels of iNKT cells and IFN-γ, which might be related with more advanced pTNM stage and more vascular invasion.

**Conclusions:**

Combination of intratumoral iNKT cells and IFN-γ is a promising independent predictor for recurrence and survival in HCC, which has a better power to predict HCC patients’ outcome compared with intratumoral iNKT cells or IFN-γ alone.

## Introduction

Hepatocellular carcinoma (HCC) ranks fifth in frequency worldwide among all malignancies and is the third leading cause of cancer mortality [1]. Although hepatectomy is the best method to provide long-term survival for HCC patients, high postoperative recurrence rate is still a major problem [2]. Hence, investigation of predictive factors for HCC prognosis will help to screen high-risk factors influencing postoperative recurrence and develop better strategies for HCC management. Recently, it has been reported that biologic behaviors of HCC are associated with a unique immune response signature [3], increased CD4(+)CD25(+)Foxp3(+) regulatory T cells (Treg) may promote hepatitis B virus-related HCC progression [4], and intratumoral balance of regulatory and cytotoxic T cells is a promising independent predictor for recurrence and survival [5], [6]. In addition, adjuvant immunotherapy might have potential to prevent postoperative recurrence [7]. Thus,it is very important to investigate immune response of HCC for better understanding of the mechanism underlying HCC metastasis and for prevention of recurrence after curative resection.

Lymphocytes are important components of liver which is considered as an immunologic organ [8]. Natural killer T (NKT) cells are one of the richest sources for hepatic lymphocytes [8]–[10], however, with the limitations of existing methodology, only a few studies of intrahepatic NKT cells in liver immunity, especially in HCC immune response, have been reported. Invariant NKT (iNKT) cells, which have a highly restricted T-cell receptor (TCR) repertoire encoded by Vα24 Jα18 paired with a set of Vβ chains in human, make up 90%–95% of total NKT cells and have been intensely investigated in most studies about NKT cells [11], [12], [13]. The expression of gene TRAV10, encoding the Vα24 domain of the T-cell receptor that characterizes iNKT cells, can be used as a specific marker of iNKT cells [14]. A hallmark of iNKT cells is the capacity to rapidly produce a mixture of T helper type 1 (Th1,i.e. IFN-γ) and T helper type 2 (Th2, i.e. IL-4) cytokines upon TCR engagement [9], [15]–[18]. Recent studies have proposed iNKT cells as type I NKT cells that enhance antitumor responses, while type II NKT cells suppress these responses [19]. The anti-tumor immunity of iNKT cells depends on the production of IFN-γ from themselves. Consistent with the fact that iNKT cells enhance tumor immunosurveillance, a positive correlation between iNKT cells and tumor prognosis has been observed in head and neck squamous cell carcinoma [20], colorectal cancer [21] and neuroblastoma patients [22]. Although cumulative studies indicate that NKT cells contribute to the pathogenesis of cirrhosis by expressing a set of cytokines involved in the progression of fibrosis, a few studies of NKT cells in HCC have been reported [23], however, intratumoral NKT cells remain potential to kill HCC cells [24]. Adoptive transfer of NKT cells exposed ex vivo to HCC-derived antigens loaded on dendritic cells (DC) can inhibit the growth of mouse HCC in vivo [25]. CD1d-mediated stimulation of NKT cells selectively activates hepatic natural killer cells to eliminate experimentally disseminated hepatoma cells in murine liver [26]. But there is no report about iNKT cells as prognostic factor for HCC recurrence and survival.

The liver, as an immune-privileged organ, has different types and function of lymphocytes compared with that in peripheral circulation, such as CD4+ iNKT cells that secret Th2 cytokines and increase in frequency in HCC patients [27]. The fate and polarization of NKT lymphocyte might be influenced by host microenvironment signaling [28]. The imbalances of proinflammatory Th1 and anti-inflammatory Th2 cytokines in the microenvironment may play a prominent role in modulating HCC tumor progression and metastasis [29]. In the present study, we selected genes TRAV10 and IFN-γ (representative Th1 cytokines gene) as target genes and analyzed these genes’ mRNA expression using quantitative real-time polymerase chain reaction (qRT-PCR). We demonstrated that either intratumoral iNKT cells and IFN-γalone or their combination was a promising independent prognostic factor for HCC recurrence and survival, however, combination of these two parameters has a better power to predict HCC patients’ outcome.

## Patients and Methods

### Patients and Specimens

224 patients receiving hepatectomy in Zhongshan hospital were randomly retrieved from a prospectively collected database as previously described [5]. Meanwhile, a normal liver tissue pool from 10 healthy liver donors was constructed as the calibrator for qRT-PCR reactions. Tumor stage was determined according to the International Union Against Cancer TNM classification system [30]. The histological grade of tumor differentiation was assigned by the Edmondson grading system. Liver function was assessed by Child-Pugh score system. The pathologic features of all the cases were re-reviewed by a skillful pathologist who had no idea about the original pathology reports. The study was approved by the Zhongshan Hospital Research Ethics Committee. Informed consent was obtained according to the committee’s regulations.

### Follow-up and Postoperative Treatment

All patients were observed with a median observation time of 28.0 months (range, 1.5 to 83.0 months). Follow-up procedures and treatment modalities after relapse were administered according to a uniform guideline as previously described [5], [31]. Overall survival (OS) time was defined as the interval between the dates of surgery and death; the data were censored at the last follow-up for living patients. Recurrence-free survival (RFS) time was defined as the interval between the dates of surgery and the first confirmed relapse, otherwise the data were censored on the date of death or the last follow-up.

### qRT-PCR

Samples were collected and snap-frozen in liquid nitrogen immediately following surgical removal of the tissue in the operating room. Adjacent nontumorous liver tissue was always derived from the same segment of the HCC nodular with a free margin from the tumor tissue. Expression of the genes encoding Vα24 (TRAV10) and IFN-γ were detected by qRT-PCR [32]. Hypoxanthine phosphoribosyltransferase 1 (HPRT1) and TATA box binding protein (TBP) were used as housekeeping genes (HKGs) as described previously [33], [34]. Primers for these genes were listed in Table 1. For data analysis the 2^−ΔΔCt^ method was used [35]. The fold-change in the target genes, normalized to average HKGs and calibrated to the expression of the normal liver pool, was calculated for each sample.

**Table 1:** Primer sequence for qRT-PCR.

Target mRNA	Primer sequence	Annealing temperature (°C)	Fragment amplified (bp)
TRAV10	F:5′AGAAAGGACGAATAAGTGCCA3′ R:5′CAGGGTCAGGGTTCTGGATA3′	60	186
IFN-γ	F:5′GCAGGTCATTCAGATGTAGCGG3′ R:5′TCATGTATTGCTTTGCGTTGGA3′	60	287
HPRT1	F:5′CCTGGCGTCGTGATTAGTG3′ R:5′CAGAGGGCTACAATGTGATGG3′	60	182
TBP	F:5′ACCACTCCACTGTATCCCTCC3′ R:5′CTGTTCTTCACTCTTGGCTCCT3′	60	285

Note. TRAV10, T cell receptor alpha variable 10; IFN-γ, interferon gamma; HPRT1, Hypoxanthine phosphoribosyltransferase 1;TBP, TATA box binding protein.

### Statistical Analysis

X-tile software [36] was used to find the optimal cut-off point for the level of TRAV10 and IFN-γ mRNA expression on the basis of overall survival. Statistical analyses were performed with SPSS 15.0 software (SPSS, Chicago, IL). Kaplan-Meier analysis was used to determine the survival. Log-rank test was used to compare patients’ survival between subgroups; the Cox proportional hazards regression model was used to perform univariate and multivariate analysis. Factors showing *P*<0.05 by univariate analysis were adopted when multivariate analysis was performed. The relationship between survival and each variable was summarized using hazard ratios (HR) and 95% confidence intervals (95% CI). Receiver operating characteristic (ROC) curve analysis was used to determine the predictive value of the parameters.

A secondary analysis was performed to assess the relationship among iNKT cells, IFN-γ and clinicopathologic characteristics. For the comparison of individual variables, χ^2^ tests, Fisher’s exact tests, and nonparametric tests were carried out as appropriate. All statistical tests were two-sided. A *P* value of less than 0.05 was considered to indicate statistical significance.

## Results

### Association of Intratumoral Vα24 (TRAV10) and IFN-γ mRNA with Patients’ Clinicopathological Characteristics

The relative level of iNKT Vα24 (TRAV10) and IFN-γ mRNA expression in intratumoral tissue was significantly lower than that in adjacent nontumorous liver tissue (Table S1). The HCC patients were divided into 2 groups based on the cutoff points of intratumoral Vα24 (TRAV10) and IFN-γ mRNA expression by X-tile software as previously described [36] As shown in Table 2, there were no difference between the two groups in terms of sex, age, hepatitis history, alpha-fetal protein (AFP), alanine transaminase (ALT), Child-Pugh score, liver cirrhosis, tumor size, tumor number, tumor encapsulation or tumor differentiation; only vascular invasion and tumor pTNM stage were strongly significantly associated with the level of intratumoral Vα24 (TRAV10) or IFN-γ mRNA expression, respectively. Low intratumoral Vα24 (TRAV10) or IFN-γ mRNA expression correlated with more advanced pTNM stage and more frequent vascular invasion.

**Table 2:** Clinico-pathological characteristics based on the level of intratumoral iNKT cells, intratumoral IFN-γ and combination of two factors.

Characteristic	Intratumoral iNKT cells			Intratumoral IFN-γ			Combination of intratumoral iNKT and intratumoral IFN-γ			
	Low	High	*P*	Low	High	*P*	I	II	III	*P*
**No. of patients**	86	138		154	70		79	82	63	
No.with post-recurrence	54	60		90	24		51	42	21	
No. with death	55	54		87	22		51	40	18	
**Sex**										
Male	75	117	0.697	135	57	0.223	70	70	52	0.587
Female	11	21		19	13		9	12	11	
**Age (y)**										
< = 51	47	65	0.336	79	33	0.666	42	42	28	0.565
>51	39	73		75	37		37	40	35	
**Hepatitis history**										
No	5	11	0.605	12	4	0.781	4	9	3	0.238†
Yes	81	127		142	66		75	73	60	
**AFP (µg/L)**										
>20	56	90	1.000	104	42	0.292	52	56	38	0.600
< = 20	30	48		50	28		27	26	25	
**ALT (U/L)**										
< = 40	41	77	0.272	80	38	0.774	37	47	34	0.400
>40	45	61		74	32		42	35	29	
**Child-Pugh score**										
A	85	137	1.000†	153	69	0.528†	78	82	62	0.547†
B	1	1		1	1		1	0	1	
**Liver cirrhosis**										
Yes	77	120	0.675	133	64	0.377	71	68	58	0.199
No	9	18		21	6		8	14	5	
**Tumor size (cm)**										
>5	48	62	0.131	82	28	0.083	45	40	25	0.123
< = 5	38	76		72	42		34	42	38	
**Tumor number**										
Multiple	23	32	0.632	41	14	0.319	21	22	12	0.488
Single	63	106		113	56		58	60	51	
**Tumor encapsulation**										
None	47	71	0.681	84	34	0.471	44	43	31	0.743
complete	39	67		70	36		35	39	32	
**Tumor differentiation**										
III-IV	41	60	0.582	70	31	0.886	38	35	28	0.782
I-II	45	78		84	39		41	47	35	
**Vascular invasion**										
Yes	52	52	0.001*	81	23	0.006*	48	37	19	0.001*
No	34	86		73	47		31	45	44	
**pTNM stage**										
IIIa	28	27	0.038*	47	8	0.002*	26	23	6	0.004*
I+II	58	111		107	62		53	59	57	

* Pearson χ^2^ test, ^†^Fisher’s exac tests.

Abbreviations: iNKT cells, invariant natural killer T cells; IFN-γ, interferon gamma; AFP, alpha-fetoprotein; ALT, alanine aminotransferase; TNM, tumor-node-metastasis.

### Prognostic Factors

At the time of the last follow-up, 114 patients had tumor recurrence, including 99 intrahepatic recurrences and 15 extrahepatic metastases and 109 patients had died, including 22 patients who died of liver failure without record of tumor recurrence. The 1-, 3-, 5-year OS rates were 75.9%, 52.7% and 45.7%, respectively; and the 1-, 3-, 5-year RFS rates were 62.4%, 46.0% and 42.0%, respectively.

**Figure 1:**
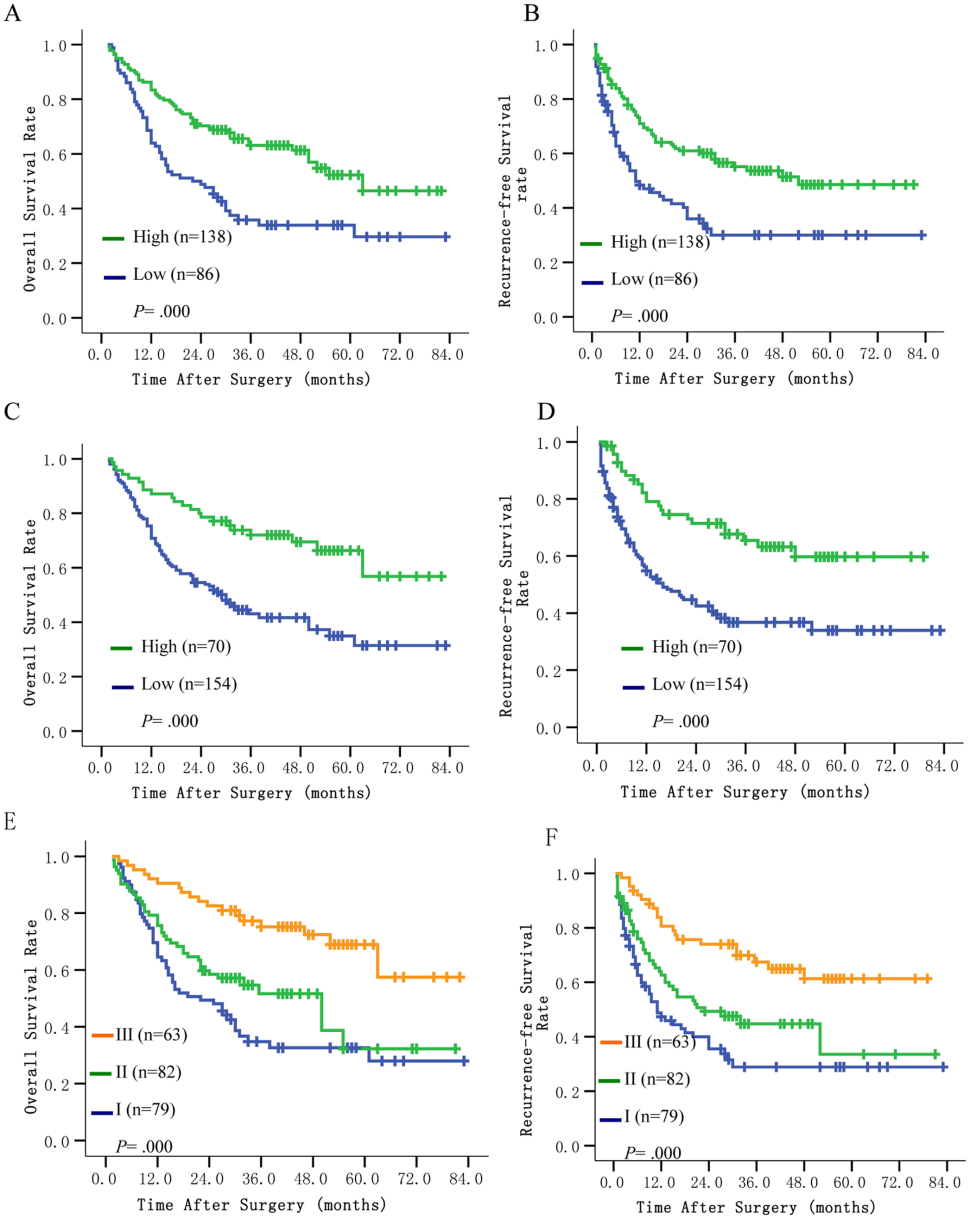
Survival curve of HCC patients. Cumulative overall and recurrence-free survival rate of HCC patients with intratumoral iNKT cells (A, B), intratumoral IFN-γ (C, D) and combination of intratumoral iNKT cells and IFN-γ (E, F). High intratumoral iNKT cells, high intratumoral IFN-γ or combination of both high was associated with prolonged overall and recurrence-free survival.

In univariate analysis, AFP, tumor size, tumor number, tumor encapsulation, presence of vascular invasion, pTNM stage, intratumoral iNKT cells, intratumoral IFN-γand combination of intratumoral iNKT cells and IFN-γ were associated with OS and RFS. Liver cirrhosis and tumor differentiation were also associated with OS (Table 3). The median OS and RFS time was prominently shorter for patients with low intratumoral iNKT cells than those with high intratumoral iNKT cells (22.0 months vs. 63.0 months and 11.0 months vs. 52.0 months, respectively; *P*<0.001 and *P*<0.001, respectively; Fig. 1A and 1B). The median OS and RFS time was significantly longer for patients with high intratumoral IFN-γ than those with low intratumoral IFN-γ (>82.0 months vs. 30.0 months and >79.0 months vs. 16.0 months, respectively; *P*<0.001 and *P*<0.001, respectively; Fig. 1C and 1D).

**Table 3:** Univariate analyses of the factors associated with survival and recurrence.

Factor	OS			RFS		
	Hazard Ratio	95%CI	*P*	Hazard Ratio	95%CI	*P*
Sex: male v female	1.125	0.652 to 1.940	0.673	1.449	0.813 to 2.583	0.209
Age: < = 51 v >51 years	1.339	0.918 to 1.952	0.129	1.307	0.904 to 1.888	0.154
Hepatitis history: no v yes	0.894	0.434 to 1.840	0.761	1.410	0.774 to 2.566	0.261
Liver cirrhosis: yes v no	2.578	1.198 to 5.549	0.015	1.646	0.883 to 3.066	0.117
ALT (U/L): < = 40 v >40	0.820	0.563 to 1.193	0.299	0.942	0.653 to 1.361	0.752
AFP(µg/L) : >20 v < = 20	1.976	1.282 to 3.046	0.002	1.907	1.260 to 2.887	0.002
Child-Pugh score: A v B	0.779	0.108 to 5,597	0.804	0.667	0.093 to 4.787	0.687
Tumor size (cm) : >5 v < = 5	2.282	1.548 to 3.363	0.000	2.152	1.477 to 3.136	0.000
Tumor number: multiple v single	1.555	1.028 to 2.353	0.037	1.737	1.162 to 2.597	0.007
Tumor encapsulation: none v complete	1.573	1.071 to 2.310	0.021	2.045	1.393 to 3.001	0.000
Tumor differentiation: III-IV v I-II	1.733	1.187 to 2.529	0.004	1.278	0.884 to 1.847	0.192
Vascular invasion: yes v no	3.673	2.457 to 5.492	0.000	3.616	2.458 to 5.318	0.000
pTNM stage: IIIa v I-II	4.621	3.122 to 6.841	0.000	5.221	3.506 to 7.776	0.000
iNKT cells: low v high	2.045	1.403 to 2.982	0.000	1.981	1.368 to 2.867	0.000
IFN-γ: low v high	2.496	1.557 to 4.001	0.000	2.444	1.553 to 3.847	0.000
Combination of iNKT and IFN-γ (I v II v III)			0.000			0.000
I v III	3.349	1.950 to 5.752	0.000	3.141	1.882 to 5.242	0.000
II v III	2.383	1.361 to 4.171	0.002	2.139	1.263 to 3.620	0.005

Abbreviations: OS, overall survival; RFS, recurrence-free survival; ALT, alanine aminotransferase; AFP, alpha-fetoprotein; TNM, tumor-node-metastasis; iNKT cells, invariant natural killer T cells; IFN-γ, interferon gamma.

In multivariate analysis, liver cirrhosis, tumor size, tumor differentiation and vascular invasion were independent risk factors for OS and tumor number, tumor encapsulation and vascular invasion were independent risk factors for RFS (Table 4). Low intratumoral iNKT cells was an independent risk factor for both OS (hazard ratio [HR] = 1.603, 95%CI: 1.091 to 2.356, *P* = 0.016) and RFS (HR = 1.786, 95%CI: 1.223 to 2.607, *P* = 0.003) (Table S2). Low intratumoral IFN-γ was also an independent risk factor for OS (HR = 2.291, 95%CI: 1.417 to 3.705, *P* = 0.001) and RFS (HR = 2.134, 95%CI: 1.349 to 3.375, *P* = 0.001) (Table S3). There was significant correlation between intratumoral iNKT cells and intratumoral IFN-γ (*r* = 0.634, *P*<0.001, Spearman’s correlation test).

**Table 4:** Multivariate analyses of the factors associated with survival and recurrence.

Factor	OS			RFS		
	Hazard Ratio	95%CI	*P*	Hazard Ratio	95%CI	*P*
Liver cirrhosis: yes v no	2.578	1.180 to 5.635	0.018			NA
AFP(µg/L):>20 v < = 20			NS			NS
Tumor size (cm): >5 v < = 5	1.562	1.025 to 2.379	0.038			NS
Tumor number: multiple v single			NS	1.570	1.036 to 2.379	0.034
Tumor encapsulation: none v complete			NS	1.533	1.025 to 2.293	0.038
Tumor differentiation: III-IV v I-II	1.631	1.110 to 2.395	0.013			NA
Vascular invasion: yes v no	2.579	1.661 to 4.005	0.000	2.950	1.973 to 4.411	0.000
pTNM stage: IIIa v I-II			NA			NA
Combination of intratumoral iNKT and IFN-γ (I v II v III)			0.001			0.001
I v III	2.784	1.603 to 4.835	0.000	2.673	1.588 to 4.499	0.000
II v III	2.481	1.410 to 4.366	0.002	1.925	1.131 to 3.278	0.016

Abbreviations: OS, overall survival; RFS, recurrence-free survival; NA, not adopted; NS, not significant; AFP, alpha-fetoprotein; TNM, tumor-node-metastasis; iNKT cells, invariant natural killer T cells; IFN-γ, interferon gamma. NOTE: We evaluated the prognostic factors that affected overall survival and recurrence-free survival using univariate analysis, and entered variables that showed statistical significance in the univariate analysis into multivariate analysis using the Cox proportional hazard regression model.

### Combination of Intratumoral iNKT Cells and IFN-γ and ROC Analysis

The combined influence of intratumoral iNKT cells and IFN-γ was evaluated. Based on the cutoff value for intratumoral iNKT cells and IFN-γ mRNA expression, patients were classified into three groups: group I (n = 79), low iNKT and low IFN-γ (neither high); group II (n = 82), high iNKT but low IFN-γ or low iNKT but high IFN-γ (either high); group III (n = 63), high iNKT and high IFN-γ (both high). Differences in both OS (*P*<0.001) and RFS (*P*<0.001) were significant among three groups (Fig. 1E and 1F). The 1-, 3-, 5-year OS rates were 69.6%, 34.8%,32.6% in group I, 75.6%, 51.6%, 32.3% in group II and 90.5%, 75.2%, 69.0% in group III. The median OS time is 22.0, 50.0 and >82.0 months in group I, group II and group III, respectively. The 1-, 3-, 5-year RFS rates were 47.3%, 28.9%, 28.9% in group I, 62.6%, 44.7%, 33.6% in group II and 80.6%, 67.5%, 61.3% in group III. The median RFS time is 11.0, 23.0 and >79.0 months in group I, group II and group III, respectively. The prognosis was the worst for HCC patients in group I, which might be related with more advanced pTNM stage and more vascular invasion in HCC (Table 2).

Combination of intratumoral iNKT cells and IFN-γ was also an independent risk factor for OS (group I vs. III, HR = 2.784, 95%CI: 1.603 to 4.835, *P* = 0.001) and RFS (group I vs. III, HR = 2.673, 95%CI: 1.588 to 4.449, *P* = 0.001) by multivariate analysis (Table 4). The area under the curve (AUC) of combination of intratumoral iNKT cells and IFN-γ was largest, compared with intratumoral iNKT cells or IFN-γ alone. The area under the curve of this combination was 0.654 (95% CI, 0.582 to 0.725; *P*<0.001) for death and 0.633 (95% CI, 0.561 to 0.706; *P* = 0.001) for recurrence (Table 5). The combination of intratumoral iNKT cells and IFN-γ had a better power to predict HCC patients’ outcomes compared with intratumoral iNKT cells or IFN-γ alone on the basis of not only hazard ratio (Table 4, Table S2 and Table S3) but also the area under the ROC curve (Table 5). Although the HR of intratumoral iNKT cells and IFN-γ were less than or comparable with HR of vascular invasion and pTNM stage and the area under ROC of vascular invasion and pTNM stage were more than the area of intratumoral iNKT cells and IFN-γ, the combination of intratumoral iNKT cells and IFN-γ was also an independent prognostic factor of poor prognosis in HCC patients with vascular invasion and pTNM stage II or III (Table S4, Table S5, Table S6 and Table S7).

**Table 5:** The predictive values of the adopted factors with pTNM stage for OS and RFS by ROC analysis.

Variables	OS			RFS		
	Area	95%CI	*P*	Area	95%CI	*P*
Vascular invasion	0.700	0.631 to 0.770	0.000	0.670	0.599 to 0.742	0.000
Combination of intratumoral iNKT cells and IFN-γ	0.654	0.582 to 0.725	0.000	0.633	0.561 to 0.706	0.001
Tumor encapsulation			NA	0.616	0.542 to 0.689	0.003
Tumor size	0.620	0.547 to 0.694	0.002			NA
Tumor number			NA	0.554	0.478 to 0.629	0.165
Liver cirrhosis	0.555	0.480 to 0.630	0.156			NA
Tumor differentiation	0.597	0.523 to 0.671	0.012			NA
pTNM stage	0.672	0.600 to 0.743	0.000	0.643	0.571 to 0.715	0.000
intratumoral iNKT cells	0.618	0.544 to 0.691	0.002	0.591	0.517 to 0.666	0.018
Intratumoral IFN-γ	0.608	0.534 to 0.682	0.005	0.604	0.530 to 0.678	0.007

Abbreviations: OS, overall survival; RFS, recurrence-free survival; NA, not adopted; TNM, tumor-node-metastasis; iNKT cells, invariant natural killer T cells; IFN-γ, interferon gamma.

## Discussion

The tumor-related factors, conventionally considered to be of prognostic value in HCC, are reasonably predictive of survival in our patient population as a whole. Our results show that either intratumoral iNKT cells or intratumoral IFN-γ is a promising factor highly predictive of OS and RFS for HCC. It also indicates that HCC patients with concurrent high intratumoral iNKT cells and IFN-γ showed significantly longer OS and RFS compared with concurrent low group, probably associated with less vascular invasion and earlier pTNM stage in this group. Vascular invasion and pTNM stage were relatively putative clinicopathologic markers of HCC invasiveness and predictive factors for recurrence in literature [37]–[40]. The combination of intratumoral iNKT cells and IFN-γ had a better power to predict HCC patients’ outcome compared with intratumoral iNKT cells or IFN-γ alone. The present results suggest that iNKT cells, which show regulatory activity on tumor, display inhibiting tumor growth and metastasis in local Th1 microenvironment. But there was different opinion about iNKT and IFN-γ as prognostic factor for HCC prognosis. Cariant et al [41] have previously reported that the higher prevalence of intratumoral iNKT cells and increased IFN-γ in adjacent nontumorous liver were associated with shorter OS and RFS, respectively. To explain difference between two studies, one of the reasons was that the related background of hepatocarcinogenesis was significantly different, more than 80% of the patients were combined with hepatitis B virus infection in this study, while the majority of the patients were hepatitis C virus infected and the hepatitis B virus infection rate was less than 10% in Carian’s study [41]. Another reason was that iNKT cells had a dual immune regulatory function, as Carian et al [41] had shown, iNKT cells producing Th2 cytokines might inhibit anti-tumor immunity through inhibiting expansion of tumor antigen-specific CD8 T cells, but iNKT cells could promote successful tumor immunosurveillance through secreting IFN-γ or under the Th1 microenvironment, which was consistent with the present study. Besides, much more cases would be investigated to further assess the prognostic value of intratumoral iNKT cells for HCC patients in Carian’s study.

IFN-γ is crucial for tumor control, and is important part of innate and adoptive immune response. This cytokine is produced by NK cells as well as CD4+ Th1 cells and CD8+ cytotoxic T lymphocyte (CTL) [42]. Locally low IFN-γ level may result from iNKT cells’ impaired function or decreased adoptive immune response. We assumed that the influence of high intratumoral iNKT cells on prognosis might probably be counteracted by simultaneously low intratumoral IFN-γ, and vice versa. In the present study, 1-, 3-, 5-year OS and RFS rates of HCC patients were significantly lower in group II than that in group III, which supports our hypothesis. Although the mechanism of interaction between intratumoral iNKT cells and IFN-γ is poorly understood, anti-metastasis effect of iNKT cells might surpass potential of pro-metastasis only under local Th1 cytokine milieu since iNKT cells secrete Th1 and Th2 cytokines at the same time.

Animal studies indicate that adoptive transfer of NKT cells exposed ex vivo to HCC-derived antigens loaded on dendritic cells (DC) can inhibit the growth of mouse HCC in vivo [25]. In some clinical trials, immunotherapy with alpha-galactosylceramide (α-GalCer) or α-GalCer–pulsed DCs can induce the activation of Vα24 NKT cells in situ [43]–[46]. A phase I study using in vitro expanded Vα24 NKT cells in patients with recurrent or advanced non-small cell lung cancer confirms that immunotherapy of activated Vα24 NKT cells administration is well tolerated with minor adverse events and in vivo immunologic responses [47]. HCC is difficult to cure due to high recurrence rate. Adjuvant immunotherapy might be a promising treatment since HCC is resistant to conventional chemotherapy. From this point of view, HCC patients undergoing curative resection may thus be candidates for immunotherapy aimed at iNKT cells activation and microenvironment IFN-γ up-regulation.

By reviewing literatures, Tachibana et al [21] have reported that intratumoral NKT cell infiltration was an independent prognostic factor for the overall and disease-free survival rate by immunohistochemistry in formalin-fixed and paraffin-embedded colorectal carcinomas tissues, but iNKT cell infiltration immunohistochemistry in HCC was not successfully carried out according to the manufacturer’s instructions with modifications [21]. Moreover approach to analysis intratumoral iNKT cell by flow cytometry might not be suitable in large sample research for the clinical outcome of HCC. Furthermore, TRAV10 gene encoding Vα24 domain of iNKT cells and its mRNA expression level has significant correlation with the number of iNKT cells [14], [22], so we measured the expression of TRAV10 gene mRNA by qRT-PCR to evaluate the prognostic value of iNKT cells for HCC patients on the basis of long-term extremely low temperature preserved specimens. In this study, we selected our institute’s cultured cells such as hepatic stellate cells (LX2), hepatocellular carcinoma cells (MHCC97L, MHCC97H, HepG2) as a negative control, the qRT-PCR did not amplify expected PCR products from these cells. In addition, real time PCR was performed using detection kits for the TRBV25-1 gene encoding the Vβ11 domain of iNKT cells as the following primer [14], F-GCACAGTTTGGGCTTTTATTTT,R-GAGAACATTCCAGAGTGATCTTC, the results was similar with the present study (data was not shown). With the improvement of iNKT cells isolation in the tissue [48], further perspective investigation is to directly detect intratumoral iNKT cells frequency or to obtain intratumoral iNKT cells. Further investigation of interaction between iNKT cells from HCC patients with different metastatic potential and IFN-γ ex vivo would give us more understanding of microenvironment influence on activity of iNKT cells.

Taken together, our results showed that combination of intratumoral iNKT cells and IFN-γ is a promising independent predictor for recurrence and survival in HCC. This combination had a better power to predict HCC patients’ outcome compared with intratumoral iNKT cells or IFN-γ alone. Adoptive transfer of iNKT cells and supply of Th1 microenvironment by IFN-γ subcutaneous injection may provide a promising adjuvant immunotherapeutic strategy for prevention of HCC after curative resection.

## Supporting Information

Table S1 **The relative level of iNKT Vα24 (TRAV10) and IFN-γ mRNA expression.**
(DOC)

Table S2 **Multivariate analyses of the factors associated with survival and recurrence.**
(DOC)

Table S3 **Multivariate analyses of the factors associated with survival and recurrence.**
(DOC)

Table S4 **Recurrence-free survival time among different groups.**
(DOC)

Table S5 **Recurrence-free survival time among different groups.**
(DOC)

Table S6 **Overall survival time among different groups.**
(DOC)

Table S7 **Overall survivor time among different groups.**
(DOC)
