# Supplementary material for: Combination of Intratumoral Invariant Natural Killer T Cells and Interferon-Gamma Is Associated with Prognosis of Hepatocellular Carcinoma after Curative Resection
Source: PLoS One. 2013 Aug 5;8(8):e70345. doi: 10.1371/journal.pone.0070345 (PMC3734128; doi:10.1371/journal.pone.0070345)
Supplement: Table S1 — The relative level of iNKT Vα24 (TRAV10) and IFN-γ mRNA expression. (DOC) [file pone.0070345.s001.doc]

**Supplementary Table S1.** The relative level of iNKT Vα24 (TRAV10) and IFN-γ mRNA expression

|  | Intratumoral tissue | | Adjacent nontumorous liver tissue | | *P* |
| --- | --- | --- | --- | --- | --- |
|  | mean | median | mean | median |  |
| TRAV10 | 1.24 | 1.15 | 1.66 | 1.78 | 0.004 |
| IFN-γ | -1.89 | -2.03 | -0.50 | -0.46 | 0.000 |

The relative changes in gene expression were calculated by the –△△Ct method as a calibrator and normalized against two housekeping genes (HPRT1 and TBP), as we previously described. Nonparametric Mann-Whitney U test was used to compare the relative level of iNKT Vα24 (TRAV10) and IFN-γ mRNA expression intratumoral tissue and adjacent nontumorous liver tissue.
