# Supplementary material for: Combination of Intratumoral Invariant Natural Killer T Cells and Interferon-Gamma Is Associated with Prognosis of Hepatocellular Carcinoma after Curative Resection
Source: PLoS One. 2013 Aug 5;8(8):e70345. doi: 10.1371/journal.pone.0070345 (PMC3734128; doi:10.1371/journal.pone.0070345)
Supplement: Table S2 — Multivariate analyses of the factors associated with survival and recurrence. (DOC) [file pone.0070345.s002.doc]

**Supplementary Table S2.** Multivariate analyses of the factors associated with survival and recurrence

| Factor | OS | | | RFS | | |
| --- | --- | --- | --- | --- | --- | --- |
|  | Hazard Ratio | 95%CI | *P* | Hazard Ratio | 95%CI | *P* |
| Liver cirrhosis: yes v no |  |  | NS |  |  | NA |
| AFP(μg/L) :>20 v <=20 |  |  | NS |  |  | NS |
| Tumor size (cm) : >5 v <=5 |  |  | NS |  |  | NS |
| Tumor number:  multiple v single |  |  | NS | 1.695 | 1.127 to 2.549 | 0.011 |
| Tumor encapsulation:  none v complete |  |  | NS | 1.507 | 1.009 to 2.251 | 0.045 |
| Tumor differentiation:  III-IV v I-II | 1.666 | 1.140 to 2.434 | 0.008 |  |  | NA |
| Vascular invasion: yes v no | 3.354 | 2.227 to 5.050 | 0.000 | 3.034 | 2.021 to 4.555 | 0.000 |
| pTNM stage: IIIa v I-II |  |  | NA |  |  | NA |
| **Intratumoral iNKT cells: low v high** | **1.603** | **1.091 to 2.356** | **0.016** | **1.786** | **1.223 to 2.607** | **0.003** |

Abbreviations: OS, overall survival; RFS, recurrence-free survival; NA, not adopted; NS, not significant; AFP, alpha-fetoprotein; TNM, tumor-node-metastasis; iNKT cells, invariant natural killer T cells.

NOTE. We evaluated the prognostic factors that affected overall survival and recurrence-free survival using univariate analysis, and entered variables that showed statistical significance in the univariate analysis into multivariate analysis using the Cox proportional hazard regression model.
