# Supplementary material for: Combination of Intratumoral Invariant Natural Killer T Cells and Interferon-Gamma Is Associated with Prognosis of Hepatocellular Carcinoma after Curative Resection
Source: PLoS One. 2013 Aug 5;8(8):e70345. doi: 10.1371/journal.pone.0070345 (PMC3734128; doi:10.1371/journal.pone.0070345)
Supplement: Table S3 — Multivariate analyses of the factors associated with survival and recurrence. (DOC) [file pone.0070345.s003.doc]

**Supplementary Table S3.** Multivariate analyses of the factors associated with survival and recurrence

| Factor | OS | | | RFS | | |
| --- | --- | --- | --- | --- | --- | --- |
|  | Hazard Ratio | 95%CI | *P* | Hazard Ratio | 95%CI | *P* |
| Liver cirrhosis: yes v no | 2.551 | 1.166 to 5.579 | 0.019 |  |  | NA |
| AFP(μg/L) :>20 v <=20 |  |  | NS | 1.518 | 0.997 to 2.311 | 0.051 |
| Tumor size (cm) :  >5 v <=5 | 1.553 | 1.020 to 2.365 | 0.040 |  |  | NS |
| Tumor number:  multiple v single |  |  | NS | 1.529 | 1.1013 to 2.307 | 0.043 |
| Tumor encapsulation:  none v complete |  |  | NS | 1.517 | 1.020 to 2.257 | 0.040 |
| Tumor differentiation: III-IV v I-II | 1.654 | 1.127 to 2.429 | 0.010 |  |  | NA |
| Vascular invasion: yes v no | 2.611 | 1.688 to 4.038 | 0.000 | 2.877 | 1.928 to 4.292 | 0.000 |
| pTNM stage: IIIa v I-II |  |  | NA |  |  | NA |
| **Intratumoral IFN-γ:**  **low v high** | **2.291** | **1.417 to 3.705** | **0.001** | **2.134** | **1.349 to 3.375** | **0.001** |

Abbreviations: OS, overall survival; RFS, recurrence-free survival; NA, not adopted; NS, not significant; AFP, alpha-fetoprotein; TNM, tumor-node-metastasis; IFN-γ, interferon gamma.

NOTE. We evaluated the prognostic factors that affected overall survival and recurrence-free survival using univariate analysis, and entered variables that showed statistical significance in the univariate analysis into multivariate analysis using the Cox proportional hazard regression model.
