# Supplementary material for: Combination of Intratumoral Invariant Natural Killer T Cells and Interferon-Gamma Is Associated with Prognosis of Hepatocellular Carcinoma after Curative Resection
Source: PLoS One. 2013 Aug 5;8(8):e70345. doi: 10.1371/journal.pone.0070345 (PMC3734128; doi:10.1371/journal.pone.0070345)
Supplement: Table S5 — Recurrence-free survival time among different groups. (DOC) [file pone.0070345.s005.doc]

**Supplementary Table S5.** Recurrence-free survival time among different groups

|  |  | Mean* | Median | *P* |
| --- | --- | --- | --- | --- |
| pTNM stage | Group |
| I | Group I | 49.7 |  | 0.382 |
|  | Group II | 49.0 |  |  |
|  | Group III | 57.9 |  |  |
| II | Group I | 23.2 | 11.0 | 0.072 |
|  | Group II | 17.9 | 23.0 |  |
|  | Group III | 46.8 |  |  |
| **III** | Group I | 6.3 | 3.0 | **0.041** |
|  | Group II | 11.6 | 6.0 |  |
|  | Group III | 22.0 | 10.0 |  |

Group I, low iNKT and low IFN-γ (neither high); Group II, high iNKT but low IFN-γ or low iNKT but high IFN-γ (either high); Group III, high iNKT and high IFN-γ (both high).

*.Estimation is limited to the largest survival time if it is censored.
