# Supplementary material for: Combination of Intratumoral Invariant Natural Killer T Cells and Interferon-Gamma Is Associated with Prognosis of Hepatocellular Carcinoma after Curative Resection
Source: PLoS One. 2013 Aug 5;8(8):e70345. doi: 10.1371/journal.pone.0070345 (PMC3734128; doi:10.1371/journal.pone.0070345)
Supplement: Table S6 — Overall survival time among different groups. (DOC) [file pone.0070345.s006.doc]

**Supplementary Table S6.** Overall survival time among different groups

|  |  | Mean* | Median | *P* |
| --- | --- | --- | --- | --- |
| Vascular invasion | Group |
| **Yes** | Group I | 20.4 | 13.0 | **0.011** |
|  | Group II | 22.7 | 13.0 |  |
|  | Group III | 52.7 | 15.0 |  |
| No | Group I | 51.6 |  | 0.137 |
|  | Group II | 57.5 |  |  |
|  | Group III | 61.4 |  |  |

Group I, low iNKT and low IFN-γ (neither high); Group II, high iNKT but low IFN-γ or low iNKT but high IFN-γ (either high); Group III, high iNKT and high IFN-γ (both high).

*.Estimation is limited to the largest survival time if it is censored.
