# Supplementary material for: Combination of Intratumoral Invariant Natural Killer T Cells and Interferon-Gamma Is Associated with Prognosis of Hepatocellular Carcinoma after Curative Resection
Source: PLoS One. 2013 Aug 5;8(8):e70345. doi: 10.1371/journal.pone.0070345 (PMC3734128; doi:10.1371/journal.pone.0070345)
Supplement: Table S7 — Overall survivor time among different groups. (DOC) [file pone.0070345.s007.doc]

**Supplementary Table S7.** Overall survivor time among different groups

|  |  | Mean* | Median | *P* |
| --- | --- | --- | --- | --- |
| pTNM stage | Group |
| I | Group I | 49.8 |  | 0.150 |
|  | Group II | 59.3 |  |  |
|  | Group III | 59.0 |  |  |
| **II** | Group I | 29.2 | 30.0 | **0.027** |
|  | Group II | 25.7 | 24.0 |  |
|  | Group III | 61.2 |  |  |
| III | Group I | 16.1 | 11.0 | 0.067 |
|  | Group II | 17.8 | 9.0 |  |
|  | Group III | 27.9 |  |  |

Group I, low iNKT and low IFN-γ (neither high); Group II, high iNKT but low IFN-γ or low iNKT but high IFN-γ (either high); Group III, high iNKT and high IFN-γ (both high).

*.Estimation is limited to the largest survival time if it is censored.
